# A Genetic Screen Identifies PRP18a, a Putative Second Step Splicing Factor Important for Alternative Splicing and a Normal Phenotype in *Arabidopsis thaliana*

**DOI:** 10.1534/g3.118.200022

**Published:** 2018-02-27

**Authors:** Tatsuo Kanno, Wen-Dar Lin, Chia-Liang Chang, Marjori Matzke, Antonius J.M. Matzke

**Affiliations:** Institute of Plant and Microbial Biology, Academia Sinica115 Taipei, Taiwan

**Keywords:** alternative splicing, *Arabidopsis thaliana*, PRP18, second step factor

## Abstract

Splicing of pre-mRNA involves two consecutive *trans*-esterification steps that take place in the spliceosome, a large dynamic ribonucleoprotein complex situated in the nucleus. In addition to core spliceosomal proteins, each catalytic step requires step-specific factors. Although the *Arabidopsis thaliana* genome encodes around 430 predicted splicing factors, functional information about these proteins is limited. In a forward genetic screen based on an alternatively-spliced *GFP* reporter gene in *Arabidopsis thaliana*, we identified a mutant impaired in putative step II factor PRP18a, which has not yet been investigated for its role in pre-mRNA splicing in plants. Step II entails cleavage at the 3′ splice site accompanied by ligation of the 5′ and 3′ exons and intron removal. In the *prp18* mutant, splicing of a U2-type intron with non-canonical AT-AC splice sites in *GFP* pre-mRNA is reduced while splicing of a canonical GT-AG intron is enhanced, resulting in decreased levels of translatable *GFP* mRNA and GFP protein. These findings suggest that wild-type PRP18a may in some cases promote splicing at weak, non-canonical splice sites. Analysis of genome-wide changes in alternative splicing in the *prp18a* mutant identified numerous cases of intron retention and a preponderance of altered 3′ splice sites, suggesting an influence of PRP18a on 3′ splice site selection. The *prp18a* mutant featured short roots on synthetic medium and small siliques, illustrating that wild-type PRP18a function is needed for a normal phenotype. Our study expands knowledge of plant splicing factors and provides foundational information and resources for further functional studies of PRP18 proteins in plants.

Messenger RNAs in eukaryotes are derived from primary transcripts (pre-mRNAs) that are subject to extensive co- and post-transcriptional processing involving 5′ cap formation, excision of introns by splicing, and 3′ cleavage and polyadenylation (Moore and Proudfoot 2009). Pre-mRNA splicing occurs in two sequential *trans*-esterification steps that take place in the spliceosome, a large dynamic ribonucleoprotein (RNP) complex present in the nucleus. The first catalytic step (step I) yields a lariat-3′ exon and a cleaved 5′ exon. The second catalytic step (step II) produces the final mRNA with ligated exons and the spliced out lariat intron (Horowitz 2012; Bertram *et al.* 2017). In constitutive splicing, the same splice sites are always used for a given intron. By contrast, alternative splicing entails variable splice site usage, which increases transcriptome and proteome diversity. Major modes of alternative splicing include intron retention (IR), exon skipping (ES) and alternative 5‘(donor) and 3′ (acceptor) splice site selection. Alternative splicing is rare in budding yeast but common in plants and animals, in which the majority of multi-intron genes undergo alternative splicing. ES is the major mode of alternative splicing in animal cells whereas IR predominates in plants (Marquez *et al.* 2012). Alternative splicing in plants is important for development and responses to the environment (Reddy *et al.* 2013; Staiger and Brown 2013; Filichkin *et al.* 2015; Deng and Cao 2017).

The spliceosomal cycle involves the step-wise assembly of at least six biochemically and/or structurally defined splicesomal complexes that vary in the composition of proteins and small nuclear (sn) RNAs (Matera and Wang 2014; Yan *et al.* 2017; Bertram *et al.* 2017). Core spliceosomal proteins include six Sm/Lsm proteins - B/B’, D1, D2, D3, E, F and G – which form a heptameric ring encircling one of five snRNAs (U1, U2, U4, U5, or U6) to create five distinct snRNPs. In addition to snRNP proteins, numerous non-snRNP proteins are dynamically associated with the spliceosome as the splicesomal cycle proceeds. As an initial step in the splicing process, the U1 and U2 snRNPs recognize the 5′ and 3′ splice sites and conserved branch point of an intron and interact to produce pre-spliceosomal complex A. The addition of a preformed U4/U6.U5 tri-snRNP to complex A yields pre-catalytic complex B. After dissociation of U1 and U4 snRNPs as well as other conformational and compositional changes to the spliceosome, the U2, U5 and U6 snRNPs act at the active sites of two sequentially-formed catalytic complexes, B* and C*, to execute the consecutive step I and II reactions, respectively (Yan *et al.* 2017; Haselbach *et al.* 2018). Catalysis is accomplished by two Mg^+2^ ions that are coordinated by conserved nucleotides in U6 snRNA (Fica *et al.*, 2013). The spliceosome can thus be described as a protein-directed metalloribozyme (Fica *et al.* 2013; Hang *et al.* 2015; Yan *et al.* 2017). The principle protein at the catalytic center is Prp8 (pre-mRNA processing factor8), a U5 snRNP component that provides a scaffold for the pre-mRNA and the U2, U5 and U6 snRNAs to ensure they are properly positioned for the splicing reactions (Hodges *et al.* 1995; Grainger and Beggs 2005; Yan *et al.*, 2017). Following the two splicing steps, the spliced mRNA and lariat intron are released from the post-spliceosomal complex P (Liu *et al.* 2017; Wilkinson *et al.* 2017). The spliceosome then dissociates, liberating individual components to assemble anew at the next intron.

Most information about the protein composition of different spliceosomal complexes has been obtained from proteomic, RNA cross-linking and structural studies performed using *Saccharomyces cerevisiae* (budding yeast) and metazoan cells (Fabrizio *et al.* 2009; Wahl *et al.* 2009; Wahl and Lührmann 2015; Will and Lührmann 2011). Results from similar proteomic and structural analyses are lacking in plants because functional spliceosomal complexes have not yet been isolated from plant cells (Reddy *et al.* 2012, 2013). Therefore, the predicted protein composition of plant spliceosomes has been largely deduced from comparative sequence analyses. These comparisons have demonstrated that the *Arabidopsis thaliana* (Arabidopsis) genome encodes approximately 430 spliceosomal proteins and splicing-related factors, the majority of which are conserved in budding yeast and metazoans (Koncz *et al.* 2012). Only a fraction of these splicing-related proteins have been studied for their contributions to splicing and plant physiology. A number of the splicing factors are encoded by duplicated genes, which can potentially diverge functionally over evolutionary time (Koncz *et al.* 2012; Reddy *et al.* 2012, 2013).

Non-snRNP proteins that associate with the spliceosome at a specific step have been identified in budding yeast and humans. Step I factors include Cwc25 and Yju2 (CWC15 and CCDC130, respectively, in humans), which both act to stabilize Prp8 in the catalytic cavity of the B* complex (Wan *et al.* 2016; Yan *et al.* 2017; Bertram *et al.* 2017). In budding yeast, step II requires four proteins not present in the spliceosome at completion of the first *trans*-esterification step. These include Prp16, a DExD/H –box RNA helicase involved in remodeling the spliceosome after step I to create catalytically-active complex C; Prp22, a DExH-box RNA helicase essential for the second *trans*-esterification step and release of the mature mRNA from the spliceosome; Slu7, a Zn finger protein; and Prp18, a protein with a unique structure unrelated to any other protein (Horowitz 2012). Slu7 and Prp18 interact with each other and contact Prp8 in complex C* to assist in docking the 3′ splice site-3′ exon into the active site (Zhang and Schwer 1997; Horowitz 2012; Ohrt *et al.* 2013; Yan *et al.* 2017; Bertram *et al.* 2017).

Arabidopsis encodes orthologs of these step I- and step II-specific factors (Koncz *et al.*, 2012), several of which have been studied previously. Of the step I proteins, a Yju2 ortholog, termed CWC16a, was identified in a genetic screen and shown to be important for splicing of a subset of pre-mRNAs (Kanno *et al.* 2017a). From the step II factors, an ortholog of Prp16, *CLUMSY VEIN* (*CUV*), was shown to influence auxin-mediated development and splicing of a selected group of pre-mRNAs (Tsugeki *et al.* 2015). A Slu7 ortholog, termed *SWELLMAP1* (*SMP1*), was found to be involved in timing of cell cycle arrest during leaf development (Clay and Nelson 2005).

PRP18 is a step II factor that has not yet been studied for its role in pre-mRNA splicing or development in plants. We report here the identification of a *prp18a* mutant in a forward genetic screen based on an alternatively-spliced *GFP* reporter gene in Arabidopsis. We describe the phenotypic features of the *prp18a* mutant and report on genome-wide differential gene expression and alternative splicing profile in the mutant.

## Materials And Methods

### Plant material

The Arabidopsis transgenic T line harboring an alternatively-spliced *GFP* reporter gene (referred to hereafter as ‘wild-type’) and the *prp18a-1* mutant derived from ethyl methane sulfonate (EMS) mutagenesis of the T line are in the Col-0 ecotype (Kanno *et al.* 2016, 2017a,b). Seeds of a *prp18b-1* T-DNA insertion mutant (SALK_024667C) were provided by the Nottingham Arabidopsis Stock Center (NASC). Plants were cultivated under long-day conditions (22-24°, 16 hr light, 8 hr dark).

### Nomenclature of plant generations

M1 mutant plants are grown from seeds treated with EMS and hence are heterozygous for EMS-induced mutations. Self-fertilization (selfing) of M1 plants produces the M2 generation, which is the first generation when a recessive mutation can be homozygous. Further selfing of M2 plants leads to generations M3, M4 and so on. Backcrossing an M2 plant with the parental T line produces the BC1 generation, in which heterozygosity of the original mutation is re-established. Selfing of BC1 plants yields the BC1F2 generation, 25% of which are again homozygous for the respective mutation. BC1F2 plants contain fewer EMS-induced mutations than the original M2 plant. Further selfing of BC1F2 plants produces generations BC1F3, BC1F4 and so forth. Crossing two lines that are homozygous for distinct mutations generates the F1 generation, in which the two mutations are heterozygous. Selfing an F1 plant yields the F2 generation, which segregates the two mutations in a Mendelian manner.

### Forward genetic screen, complementation, and phenotype analysis

Details of the forward genetic screen based on an alternatively-spliced *GFP* reporter gene in the wild-type T line have been described in previous publications (Kanno *et al.* 2016, 2017a,b). Mutagenesis was accomplished using EMS, which produces almost exclusively G/A to C/T transition mutations (Kim *et al.* 2006). Screening of mutants was carried out in the M2 generation. Mutants modified in splicing of the *GFP* pre-mRNA display either a GFP-weak (*gfw*) or Hyper-GFP (*hgf*) phenotype relative to the wild-type T line, which shows an intermediate level of fluorescence. So far we have reported five *hgf* and three *gfw* mutants retrieved from this screen (Table 1). The *gfw4-1* mutant described here was identified by the GFP-weak phenotype of M2 seedlings growing under sterile conditions on solid Murashige and Skoog (MS) medium using a Leica M165FC fluorescence stereomicroscope. A mutation in the *PRP18a* gene (At1g03140) in *gfw4-1* was identified by next generation mapping (NGM) (James *et al.* 2013) after sequencing pooled DNA isolated from at least 50 BC1F2 seedlings exhibiting a GFP-weak phenotype as described previously (Kanno *et al.* 2017a,b). Phenotypic analysis of the *prp18a-1* mutant was performed on plants of the BC1F3 generation.

**Table 1:** Mutants identified so far in forward genetic screen

*Hyper-GFP* (*hgf*) mutant	Name	AGI number	Predicted function in splicing	No. of alleles	Effect on GFP pre-mRNA splicing	Effect of mutation on development	Reference
*hgf1*	coilin;	At1g13030	a protein marker protein for Cajal bodies, which are facilitate snRNP maturation	12*	AU-AC↑	no	Kanno *et al.* 2016
*hgf2*	CWC16a	At1g25682	step I factor	2*	AU-AC↑	no	Kanno *et al.* 2017a
*hgf3*	SMU1	At1g73720	which may help to recognize spliceosomal targets for ubiquitination	1	AU-AC↑	no	Kanno *et al.* 2017a
*hgf4*	SMFa	At4g30220	a small nuclear ribonucleoprotein present in snRNPs	1	No change	no	Kanno *et al.* 2017a
*hgf5*	PRP39a	At1g04080	U1 snRNP component	5*	AU-AC↑	no	Kanno *et al.* 2017b

*GFP-weak* (*gfw)* mutant	Name	AGI number	Predicted function in splicing	No. of alleles	Effect on GFP pre-mRNA splicing	Effect of mutation on development	Reference
*gfw1*	AtRTF2	At5g58020	may contribute to ubiquitin-based regulation of the spliceosome	2	AU-AC↓, unspliced↑	embryo lethal	Sasaki *et al.*, 2015; Kanno *et al.* 2017a
*gfw2*	PRP8a	At1g80070	a U5 snRNP component that acts at the catalytic core of the spliceosome	3	AU-AC↓, unspliced↑	embryo lethal	Sasaki *et al.*, 2015; Kanno *et al.* 2017a
*gfw3*	RBM25	At1g60200	U1 snRNP component	2	AU-AC↓, unspliced↑	low seed set	Kanno *et al.* 2017b
*gfw4*	PRP18a	At1g03140	step II factor	1	AU-AC↓, GU-AG↑	short roots, small siliques	this study

The mutants obtained so far include a core spliceosomal protein (SMFa); components of the U1 (PRP39a, RBM25) and U5 (PRP8) snRNPs; step I and step II factors transiently associated with the spliceosome (CWC16a and PRP18a, respectively); putative splicing regulatory proteins (RTF2 and SMU1); and one structural protein presumed to be important for snRNP maturation (coilin). So far we have only observed developmental phenotypes with the four identified *gfw* mutations, two of which are embryo-lethal. The biological significance of these results are not yet clear.

* Further screening of the M2 population after publication of the first alleles of coilin, PRP39a and CWC16a has identified three new alleles of coilin (R9H; first intron, 3′ splice site; second intron, 5′ splice site), one new allele of PRP39a (R226*), and one new allele of CWC16a (W18*). These new unpublished alleles are included in the number of alleles shown in this table.

Complementation of the *prp18a-1* mutation was achieved by transforming the mutant with a construct containing the *PRP18a* coding sequence under the control of the 35S promoter and terminator sequences (Pietrzak *et al.* 1986). Mutant plants (BC1F3 generation) were transformed with this construct using the floral dip method (Clough and Bent 1998) and *Agrobacterium* binary vector BV-Mp*PAT*ot *Sal*I (Matzke *et al.* 2010), which confers resistance to phosphinothricin (PPT). T1 transformants were selected on solid MS medium containing 200 µg/ml cefotaxime and 20 µg/ml PPT. The presence of the *prp18a-1* mutation in complemented lines was confirmed by Sanger sequencing.

### Testing the effects of a prp18b mutation on GFP fluorescence and plant phenotype

To test whether a homozygous mutation in *PRP18b* (At1g54590), the paralog of *PRP18a*, would similarly confer a GFP-weak phenotype and phenotypic defects, we crossed the wild-type T line (*T/T*) with a homozygous *prp18b-1* T-DNA insertion mutant (*b/b*) (SALK_024667C). Self-fertilization of the F1 plants resulting from the cross (genotype *T/*-; *B/b*; the dash denotes hemizygosity for the transgenic *T* locus) generated a segregating F2 population. F2 seeds were germinated on solid MS medium and screened approximately two weeks later under a fluorescence stereomicroscope for GFP expression, which is observed with a genotype of either *T/T* or *T/*- (collectively written hereafter as *T/(T)*). A subset was transferred to soil for genotyping to identify *T/T*; *b/b* plants. *T/T;b/b* plants in soil were examined for phenotypic features during growth and reproduction. Selfed seedlings of *T/T;b/b* plants were sown on MS medium and viewed under a fluorescence stereomicroscope to assess GFP expression, and the length of seedling roots on MS medium was noted.

To investigate the viability of double homozygous mutant plants (*a/a*; *b/b*), we crossed the homozygous *prp18-1* mutant (*T/T*; *a/a*) to a *b/b* plant. Self-fertilization of the F1 plants resulting from this cross (genotype *T/*-; *A/a*; *B/b*) produced a segregating F2 population. The F2 seeds were germinated on solid MS medium and pre-screened under a fluorescence stereomicroscope for a GFP-weak phenotype (indicating a genotype of *T/(T)*; *a/a*). Selected GFP-weak F2 progeny were transferred to soil for genotyping to identify *T/(T)*; *a/a*; *b/b* plants. Primers for detecting *prp18a-1* and *prp18b-1* alleles are listed in Table S5.

### Western blotting

Western blotting to detect GFP protein was carried as described previously using total protein isolated from two week-old seedlings (BC1F3 generation) growing on solid MS medium under a 16 hr light/8 hr dark cycle at 24° (Fu *et al.* 2015; Kanno *et al.* 2016, 2017a,b). Monoclonal GFP antibodies were obtained from Roche (Cat. No. 11814 460001). Actin monoclonal antibodies were purchased from Thermo Scientific Pierce (Cat. No. MA1-774).

### Semi-quantitative RT-PCR

Semi-quantitative RT-PCR to detect *GFP* splice variants in the *prp18a-1* mutants was conducted using total RNA isolated from two week-old seedlings (BC1F3 generation) growing on solid MS medium as described above using a Plant Total RNA Miniprep kit (GeneMark, Taiwan) according to a published procedure (Sasaki *et al.* 2015; Kanno *et al.* 2017a,b). Primers for GFP and actin are listed in Table S5.

### RNA-sequencing (RNA-seq)

Total RNA was isolated from two-week-old seedlings (cultivated on MS medium as described above) of the original inbred *prp18a-1* mutant, the BC1F3 generation of the *prp18a-1* mutant, and the wild-type T line. Library preparation and RNA-seq performed out (biological triplicates for each sample) as described previously (Sasaki *et al.* 2015; Kanno *et al.* 2016). Whole genome re-sequencing on the *prp18a-1* mutant was conducted to detect remaining EMS-induced second-site mutations that alter splice sites, which were then removed from the analysis of alternative splicing.

RNA-seq reads were mapped to the TAIR10 genome using the following two-step approach. Reads were mapped to the TAIR10 transcriptome using Bowtie2 (Langmead and Salzberg 2012), only alignments of read pairs that were mapped to the same transcript with high identity (>95%) and at least 4bp exact matches in both ends were accepted. Rest reads were mapped to the TAIR10 genome using BLAT (Kent 2002), where alignments with blocks shorter than 8bps were removed. Read counts were computed using RackJ (http://rackj.sourceforge.net/) and normalized into log-count-per-million (logCPM) using the TMM method (Robinson and Oshlack 2010) and the voom method (Law *et al.* 2014). logCPM values were then transformed into RPKM values. Differentially expressed genes were identified using *t*-tests on aforementioned RPKM values if the p-value was less than 0.01 and the fold-change was greater than or equal to 2.

To detect the preference of retaining an intron, its intron retention ratio was computed as the average read depth of this intron divided by the average read depth of neighboring exons, and its intron retention ratios in mutant replicates were compared to those in wild-type controls using *t*-test. In this approach, the underlying null hypothesis assumes that the chance of retaining the intron is the same in both samples, and a significant p-value indicates that the chance is not the same, *i.e.*, a preference of intron retention in one sample. In this study, we classified an intron as intron retention (IR) or more efficient splicing (MES) if its *t*-test p-value was less than 0.01 and the average intron retention ratio in mutant replicates was two times higher or lower than that in wild-type controls, respectively.

To detect the preference of an exon skipping event or an alternative donor/acceptor event, an approach similar to that for introns was applied. The alternative splicing (AS) ratio was computed as the number of supporting reads of the AS event divided by the number of non-supporting reads of the AS event (splice reads involving one skipped exon for exon skipping events, and splice reads spanning the same exon pair but with other splicing junctions for alternative donor/acceptor events), and log-AS ratios in mutant replicates were compared to those in wild-type controls using *t*-test. For further confirmation of the AS event, its expression ratios were also computed as supporting read counts divided by unique read counts of its gene, and applied log-expression ratios to *t*-test. Finally, an exon skipping event or an alternative donor/acceptor event was reported if both p-values were less than 0.01, and it was classified as enhanced or reduced if the average expression ratio in mutant replicates was two times higher or lower than that in wild-type controls, respectively.

### Reagent and data availability

Seeds of the homozygous T line are available from the Arabidopsis Biological Resource Center, (ABRC) Ohio State University, under the stock number CS69640. Seeds of the *prp18a-1* mutant will be deposited at ABRC and are currently available on request from the Matzke lab.RNA-sequencing data for the prp18 mutants reps 1-6 and the wild-type T control samples for the *prp18* mutants reps 4-6 (T wild type reps 1-3) as well as whole genome re-sequencing data for the prp18a-1 mutant are available under Sequence Read Archive (SRA) accession number SRP119240. The wild-type T controls for the *prp18* mutant reps 1-3 are under SRA accession numbers SRP093582 (ST biological replicates 4-5) and SRP119240 (T wild type reps 4-6).

## Results

### Identification of a prp18a mutant in forward genetic screen

**Figure 1:**
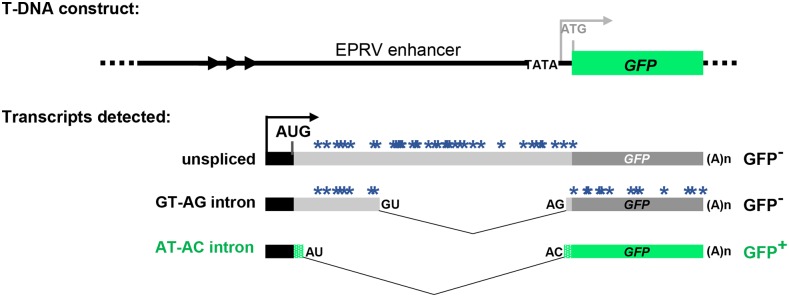
Schematic drawing of alternatively-spliced *GFP* reporter gene. Top: The T-DNA original construct introduced into Arabidopsis contained a *GFP* reporter gene under the transcriptional control of a minimal promoter (TATA) and upstream viral (EPRV) enhancer element. However, analysis of the wild-type T line revealed that neither the minimal promoter nor the downstream ATG initiation codon (gray letters) is used. Bottom: The T line analysis indicated that transcription of *GFP* pre-mRNA initiates at an upstream promoter (black bar and arrow) to generate three alternative splice variants that comprise part of the enhancer region (Kanno *et al.* 2008). These variants include a long unspliced transcript, a middle-length transcript arising from splicing of a canonical GT-AG intron, and a short transcript resulting from splicing a U2-type intron with non-canonical AT-AC splice sites, which are generally considered inefficient splice sites (Crotti *et al.* 2007). Because the unspliced and GU-AG transcripts contain a number of premature termination codons (blue asterisks), only the AU-AC transcript can be translated into GFP protein. The actual coding sequence of GFP protein (green bars) contains a unique 27 amino acid extension (short stippled green bars) relative to standard GFP (Fu *et al.* 2015; Kanno *et al.* 2016). Arrowheads designate a short tandem repeat upstream of the promoter. The black AUG denotes the major translation initiation codon. The distance between the 3′ splice sites for the GT-AG and AT-AC introns is only 3 nt; the non-canonical AC is on the outside (Kanno *et al.* 2016, 2017a,b).

In the wild-type T line used in this study, *GFP* pre-mRNA is alternatively spliced to yield three main splice variants: a long unspliced transcript, a mid-length transcript arising from splicing a canonical GT-AG intron, and a short transcript resulting from splicing a U2-type intron with non-canonical AT-AC splice sites (Sasaki *et al.* 2015). Because the unspliced and GU-AG transcripts contain a number of premature termination codons, only the AU-AC transcript represents a *bona fide GFP* mRNA that can be translated into GFP protein (Figure 1). The three *GFP* transcripts are present in a balanced ratio in the wild-type T line, which displays an intermediate level of GFP fluorescence (Kanno *et al.* 2017a,b). Our working hypothesis is that mutations in genes encoding splicing factors will modify the ratio of the three transcripts, leading to either elevated or lowered *GFP* mRNA levels. These changes will result, respectively, in either a Hyper-GFP (*hgf*) or GFP-weak (gfw) phenotype compared to the intermediate level of the wild-type T line (Kanno *et al.* 2016, 2017a,b). Results from the screen so far support this hypothesis: we have identified five *hgf* mutants and three *gfw* mutants that vary in the splicing pattern of *GFP* pre-mRNA (Table 1). Here we report the finding of a fourth mutant, *gfw4*, in the GFP-weak category.

**Figure 2:**
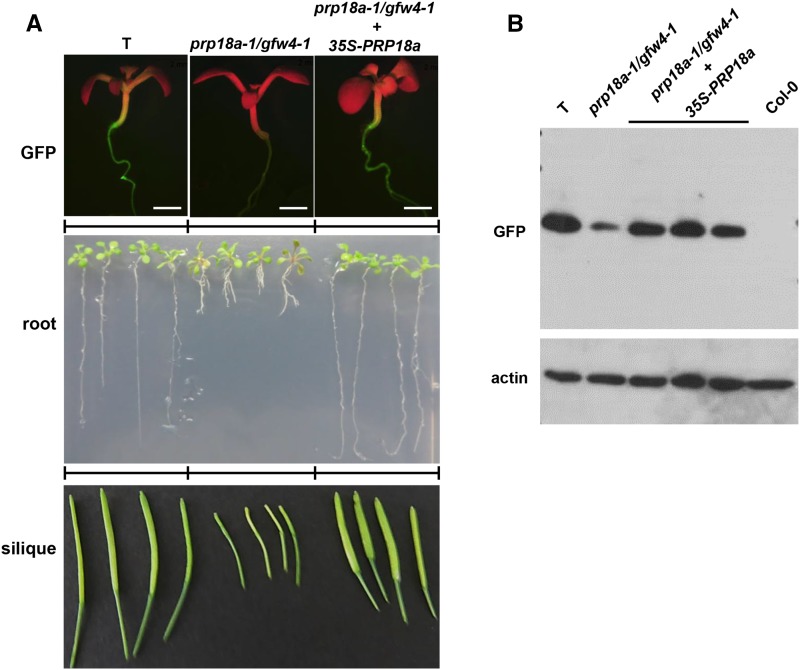
Phenotypic analysis of the *prp18a-1/gfw4-1* mutant and complemented line. **A.** GFP fluorescence in seedlings (top), root growth of seedlings on solid MS medium (middle) and developing siliques (bottom) of the wild-type T line, the *prp18-a-1/gfw4-1* mutant and the *prp18a-1/gfw4/1* mutant complemented with a *PRP18a* transgene under the control of the 35S promoter. B. Western blot analysis of GFP protein in the wild-type T line, *prp18a-1/gfw4-1* mutant and three complemented lines. Non-transgenic Col-0 is shown as a negative control. The top panel was probed with an antibody to GFP protein. The bottom panel shows the same blot re-probed with an antibody to actin as a loading control.

**Figure 3:**
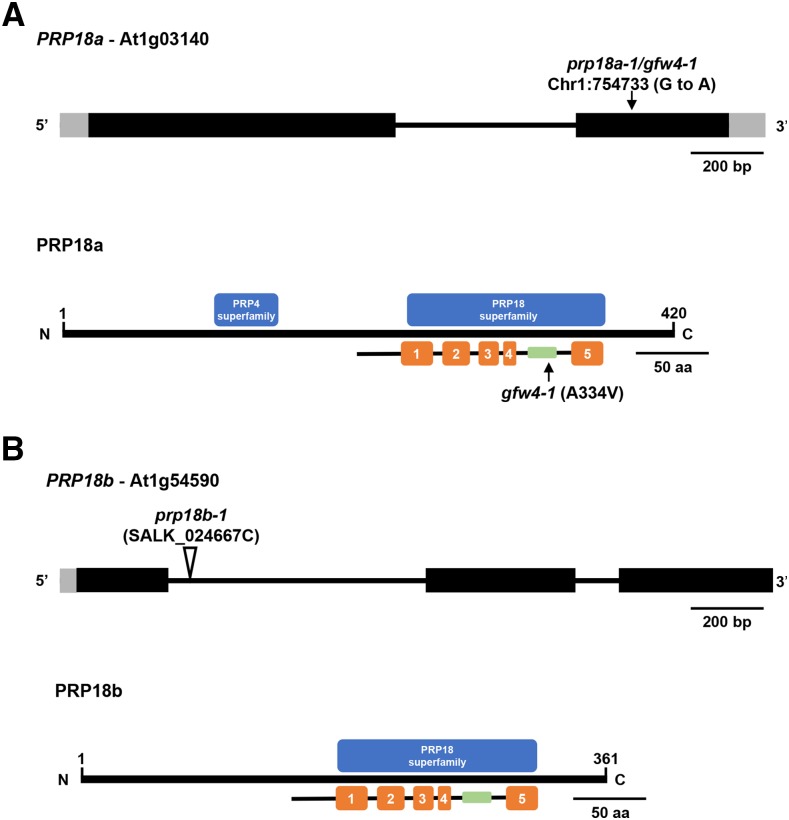
Gene structures, positions of mutations, and protein domains of *PRP18* paralogs in Arabidopsis. (**A**) The *PRP18a* gene (At1g03140) contains one intron (thin black bar) and encodes a protein 420 amino acids in length. The G to A transition mutation at position 754733 on chromosome 1 in the *prp18a-1/gfw4-1* mutant is indicated (top). The PRP18a protein contains a PRP18 domain, which comprises five α-helices (orange bars). A highly conserved loop (green bar), which is important for PRP18 function, is between the fourth and fifth helices. The *prp18a-1/gfw4-1* mutation leads to an alanine to valine substitution at position 334 within this conserved loop. This alanine residue is highly conserved in various plant species (Figure S1). PRP18a also contains a PRP4 domain of unknown function. Intact PRP4 proteins are components of U4/U6 and U4/U6.U5 snRNPs. PRP18 family proteins are conserved in yeasts and metazoans (Figure S2). (B) The *PRP18b* gene (At1g54590) contains two introns and encodes a protein 361 amino acids in length. Much of the length difference between PRP18a and PRP18b is due to missing N-terminal sequences in PRP18b (Figure S1). The PRP18b protein contains a PRP18 domain but not a recognizable PRP4 domain. The *prp18b-1* allele contains a T-DNA insertion in the first intron.

The *gfw4* mutant was identified in an EMS-mutagenized population by the GFP-weak phenotype of M2 seedlings grown on MS medium (Figure 2A). A reduction of GFP protein in the *gfw4* mutant was confirmed by Western blotting using an antibody to GFP (Figure 2B). The causal mutation in the *gfw4* mutant was determined by next generation mapping (James *et al.* 2013) to be a G to A transition mutation in the coding region of the gene encoding PRP18a (At1g03140), a putative step II splicing factor that is 420 amino acids in length. The mutation leads to a substitution of an alanine by valine at position 334 in the PRP18a protein (Figure 3A). As the first mutation reported for *PRP18a*, we designated this allele *prp18a-1/gfw4-1*.

PRP18 is an evolutionarily conserved protein, with orthologs found in other plants, yeasts and metazoans (Figure S1 and Figure S2). Prp18 proteins typically have five alpha-helices separated by four loops, with a highly conserved loop between the fourth and fifth helices (Bacíková and Horowitz 2002; Annamalai 2011). The A334V mutation we identified in the *prp18a-1* mutant is in this highly conserved loop region (Figure 3). PRP18a contains two putative nuclear localization signals (Annamalai 2011) and is predicted to be a nuclear protein (http://suba.live/).

Complementation of the *prp18a-1* mutant with a construct comprising the wild-type *PRP18a* coding sequence under the transcriptional control of the 35S promoter and terminator sequences (35Spro-*PRP18a*-35Ster) restored an intermediate level of GFP fluorescence and increased GFP protein abundance to that observed in wild-type T seedlings (Figure 2A, top and B, respectively). These findings confirm that the *prp18a-1* mutation is responsible for the GFP-weak phenotype of the *gfw4-1* mutant. The *prp18a*-1 mutation is recessive, as indicated by the intermediate level of GFP fluorescence in BC1 progeny obtained by backcrossing the mutant to a wild-type T plant.

### Phenotypic features of prp18a mutant

The phenotype of the *prp18a-1* mutant compared to the wild-type T line was monitored in the BC1F3 generation, which has a reduced number of mutations relative to the original mutant. Mutant *prp18a-1* seedlings germinated on solid MS medium at approximately the same time as wild-type seedlings; however, after two weeks of growth, the roots were noticeably shorter in the mutant than in wild-type seedlings (Figure 2A, middle). Despite a tendency to be somewhat bushy and flimsy, adult plants of the *prp18a-1* mutant generally resembled wild-type plants in terms of stature and overall appearance (Figure S3). The *prp18a-1* mutant flowered around the same time as the wild-type, but the siliques of the mutant developed more slowly and were smaller relative to wild-type (Figure 2A, bottom). The short root and small silique phenotypes of the mutant were both complemented by a 35Spro-*PRP18a*-35Ster transgene (Figure 2C), demonstrating that these aberrant characteristics were indeed due to the *prp18a-1* mutation.

### Detection of GFP splicing variants by RT-PCR

**Figure 4:**
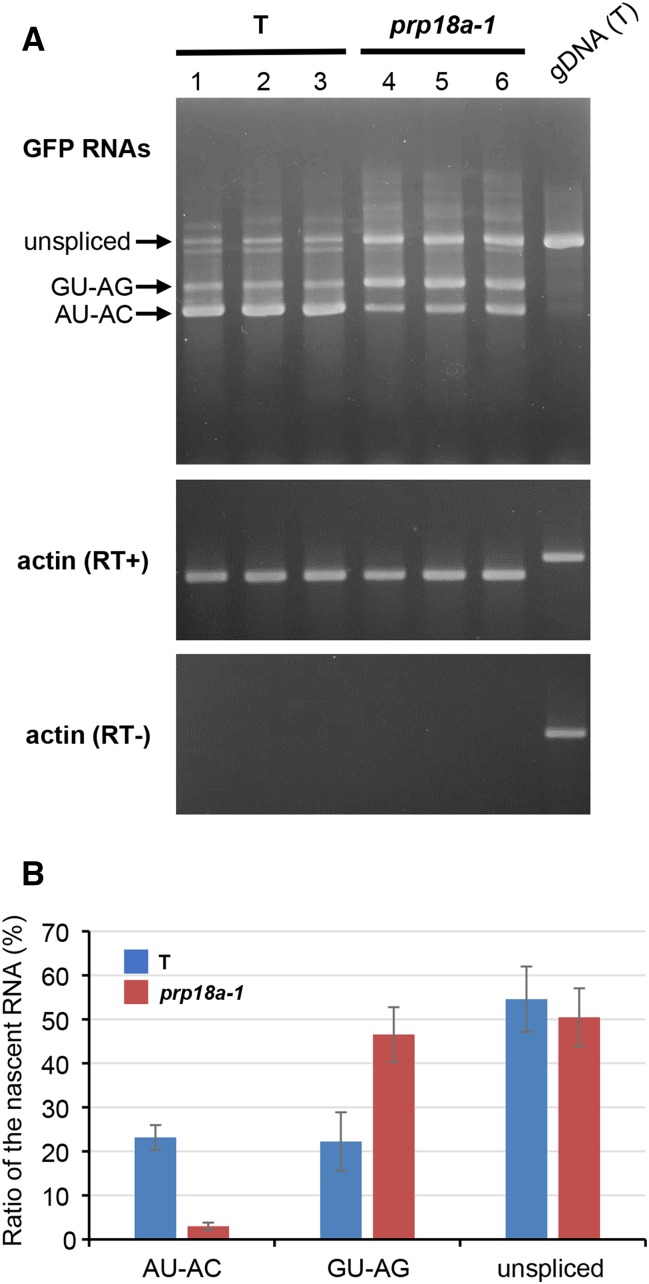
RT-PCR analysis of *GFP* splice variants in *prp18a-1* mutants. (**A**) Semi-quantitative RT-PCR was used to assess the accumulation of unspliced *GFP* transcript and two splice variants (resulting from splicing the canonical GT-AG and non-canonical AT-AC introns, respectively) in triplicate samples of the *prp18a-1* mutant and the wild-type T line. Actin is shown as a constitutively expressed control. RT- and RT+ panels show experiments with and without reverse transcriptase, respectively. gDNA (T), genomic DNA isolated from T line. (B) The percentages of three major *GFP* RNA splice variants as determined from an analysis of RNA-seq data (Table S2). The average of five biological replicates is shown. The amount of total *GFP* transcripts did not change significantly in *prp18a-1* mutants.

Semi-quantitative RT-PCR was used to examine the splicing pattern of *GFP* pre-mRNA in the *prp18a-1* mutant. The level of the translatable AU-AC transcript was reduced, consistent with the GFP-weak phenotype of the *prp18a-1* mutant. However, the level of untranslatable GU-AG transcript appeared to be increased in *prp18a-1* relative to the wild-type T line (Figure 4A). This is a unique splicing pattern of *GFP* pre-mRNA that has not been observed in any other mutant retrieved so far in the screen (Table 1).

### RNA-sequencing (RNA-seq) analysis

To analyze more comprehensively the effect of a homozygous *prp18a-1* mutation on alternative splicing, we carried out RNA-seq using total RNA isolated from two week-old seedlings of the original inbred *prp18a-1* mutant (M4 generation), BC1 F3 seedlings of the *prp18a-1* mutant, and the wild-type T-line. All samples were run in biological triplicate. Only changes that were statistically significant in both the original mutant and BC1F3 plants are considered here.

The RNA-seq data confirmed the RT-PCR findings on alternative splicing of *GFP* pre-mRNA: the percentage of translatable AU-AC transcript decreased from 23 to 3% of the total in the *prp18a-1* mutant whereas the percentage of the untranslatable GU-AG transcript increased from 22 to 47%. The percentage of unspliced transcript remained roughly the same in the *prp18a-1* mutant and wild-type T line (51% and 55%, respectively) (Figure 4B).

A summary of the results of a genome-wide analysis of differentially expressed genes (DEGs) and alternative splicing is shown in Table 2 (Details available in Table S1, Table S2, Table S3 and Table S4). The number of DEGs was relatively modest (174 total), with more up-regulated genes (160) than down-regulated genes (14). No splicing factors were found in either category.

**Table 2:** DEGs and alternative splicing events in the *prp18a-1* mutant

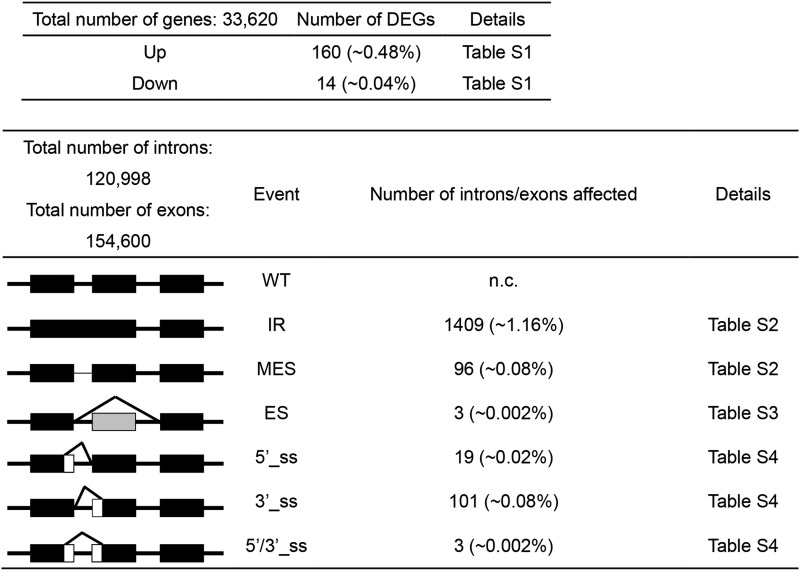

The major modes of alternative splicing are shown at the left. Abbreviations: WT, wild-type, IR, intron retention; MES, more efficient splicing; ES, exon skipping; 5′ or 3′_ss, change in 5′ splice site donor or 3′ splice site acceptor; 5′/3′_ss. Change in both 5′ and 3′ splice sites. Numbers in parentheses indicate the percentage of total introns or exons affected. Details of the RNA-seq analysis can be viewed in the indicated supporting tables.

The largest number of alternative splicing events occurred in the category of intron retention (IR), which affected 1409 introns (in 1322 genes). By contrast, only 96 cases (in 89 genes) of more efficient splicing (MES), which involves a decrease in the level of a partially retained intron in wild-type plants, were observed (Table 2). Of the IR events, fifteen involved splicing factors, including PRP39a, PRP40b and BRR2C (Table 3). These three factors were also found to undergo changes in alternative splicing in *prp39a* mutants, which were identified in the same genetic screen described here (Table 1) (Kanno *et al.* 2017b). Only three instances of exon skipping were detected. Regarding altered splice site selection, there were approximately five times more cases of altered 3′ splice sites (101) than 5′ splice sites (19) in the *prp18a-1* mutant (Table 2)

**Table 3:** Splicing factors in the intron retention (IR) list in the *prp18a-1* mutant

Gene	Protein	Predicted function in splicing^1^	Reference
At1g04080	PRP39a	U1 snRNP	Kanno *et al.* 2017b
At1g14650	SF3a120, SAP114	17S U2 snRNP	
At1g60900	U2AF65B	Splice site selection	Park *et al.* 2017
At1g76860	Lsm3B	small nuclear ribonucleoprotein	Golisz *et al.* 2013
At2g16940	fSAP59, CC1-like splicing factor	recruited prior to B*	
At2g29210	SRm160	SR-related protein	
At2g40650	PRP38	U4/U6.U5 tri-snRNP	
At2g43370	U11/U12-35K	U11/U12-specific	Lorkovic *et al.* 2005 (Table 1)
At3g13200	cwc15	Putative splicing factor	http://www.arabidopsis.org/index.jsp
At3g19670	PRP40B	U1 snRNP-related	Kang *et al.*, 2009; Kanno *et al.* 2017b
At3g49430	SR34A	SR protein, putative pre-mRNA splicing factor SF2	Barta *et al.* 2008, 2010
At4g02430	SR34B	SR protein	Barta *et al.*, 2008, 2010
At4g14342	SF3B10a splicing factor 3B subunit 5	17S U2 snRNP	Lorkovic *et al.* 2005
At5g08290	Yellow leaf-specific gene 8 (YLS8)	U5 snRNP	
At5g61140	BRR2C	U5 snRNP helicase	Mahrez *et al.*, 2016; Kanno *et al.* 2017b

For complete list of IR events, see Table S2.

1 Except for At3g13200, predicted functions taken from Table S1 of Koncz *et al.*, 2012. References in which a factor is mentioned specifically in a publication are listed (reference information taken from http://www.arabidopsis.org/index.jsp).

### Tests of prp18b

In Arabidopsis, *PRP18a* has an annotated paralog, *PRP18b* (At1g54590) (http://www.arabidopsis.org/). The *PRP18b* gene is predicted to encode a protein that is 361 amino acids in length (Figure 3B). However, PRP18b does not appear to be transcribed in two week-old seedlings, which represent the developmental stage analyzed in this study, nor was a transcript detected in an RNA-seq analysis of floral material (Figure S4). Nevertheless we introduced a T-DNA insertion mutation of *PRP18b* into both the wild-type T line and the *prp18a-1* mutant, and examined the *prp18b-1* single mutant and the *prp18a-1 prp18b-1* double mutant for GFP fluorescence, *GFP* pre-mRNA splicing and plant phenotype. By these criteria, the *prp18b* single mutant appeared identical to wild-type plants and the *prp18a prp18b* double mutant, which was viable, resembled the *prp18a-1* single mutant (Figure S4).

## Discussion

In a forward genetic screen for mutants showing modified splicing of an alternatively-spliced *GFP* reporter gene in Arabidopsis, we identified a mutation in the gene encoding PRP18a, an evolutionarily conserved, putative step II splicing factor. Prp18 was first identified in a forward screen in budding yeast for temperature-sensitive splicing mutations (Vijayraghavan *et al.* 1989) and subsequently shown biochemically to participate in step II of splicing in that organism (Horowitz and Abelson 1993). A recent structural analysis using cryo-electron microscopy (cryo-EM) in budding yeast revealed that Prp18 is enriched in the step II catalytic complex C*, where it has direct interactions with PRP8 and U5 snRNA at the active site (Yan *et al.* 2017). The mutation we identified leads to an amino acid substitution in a highly conserved loop between helices 4 and 5 of the PRP18a protein. Mutations in this loop region, which is the most evolutionarily conserved part of the PRP18 protein, are known from budding yeast to result in a deficiency in the step II reaction, likely because of disrupted interactions with PRP8 and RNA elements at the catalytic site (Yan *et al.* 2017). The location of the *prp18a-1* mutation, which affects an alanine residue that is conserved in the plant and yeast species examined, is consistent with a loss of function (or partial loss of function) allele. The nature of the *prp18a- 1* mutation suggests that Arabidopsis PRP18A acts similarly to budding yeast Prp18 in the step II reaction. However, confirmation of this proposal awaits the development of methods to isolate plant spliceosomal complexes that can be used to analyze splicing reactions *in vitro*.

PRP18 is not essential in budding yeast but cells lacking this protein grow slowly and are temperature sensitive (Bacíková and Horowitz 2002). Whether PRP18a is essential in Arabidopsis is unclear. The *prp18a-1* mutation, which may only lead to partial loss of function, is not lethal but it confers an aberrant phenotype most visible in short roots of seedlings on solid MS medium and small siliques. Although the molecular basis of this phenotype remains to be explored, the findings affirm the necessity of wild-type PRP18a function throughout plant growth and development.

### Effect of prp18a-1 mutation on pre-mRNA splicing

There are several differences in how the *prp18a-1* mutation affects splicing compared to mutations in other splicing factors identified so far in this screen. First, the splicing pattern of *GFP* pre-mRNA in *prp18a-1*, which features decreased splicing of the non-canonical AT-AC intron and increased splicing of the canonical GT-AG intron, is unique. Other *gfw* mutants generally show reduced splicing efficiency of both the AT-AC and GT-AG introns together with increased levels of unspliced *GFP* transcript. Conversely, the *hgf* mutants display increased splicing of the AT-AC intron and decreased splicing of the GT-AG intron accompanied by generally reduced accumulation of the unspliced transcript. One interpretation of these results, which does not imply a specific mechanism, is that wild-type PRP18a protein enhances splicing at non-canonical or inefficient splice sites whereas the HGF factors act in an opposite manner to normally repress splicing at non-canonical and inefficient splice sites. In budding yeast, Prp18 has been reported to suppress splicing at non-canonical sites (Kawashima *et al.* 2014). Although this finding appears contrary to ours, it nevertheless suggests a role for PRP18 in discriminating between strong and weak splice sites.

A second difference, which was revealed in the genome-wide analysis of splicing, is that the *prp18a-1* mutation affects splicing of a higher proportion of splicing factors than other mutants identified in the screen. The significance of this finding is unclear at present but it may suggest that PRP18a is deeply embedded in cross-regulatory networks involving multiple splicing factors, which are thought to coordinate responses of the spliceosome to developmental and environmental cues (Barta *et al.* 2008).

A third notable difference in splicing in the *prp18a-1* mutant compared to other mutants retrieved in the screen is the preponderance of 3′ splice site changes. In *prp18a-1*, the number of 3′ splice site alterations exceeds 5′ splice site alterations by fivefold. We did not observe such a substantial skew toward either 5′ or 3′ splice site changes in any other mutant, which show at most a twofold difference between changes at the two splice sites (Kanno *et al.* 2017a,b). The predominance of 3′ splice site alterations in the *prp18a-1* mutant might reflect the participation of PRP18a specifically in step II of splicing and potentially 3′ splice site selection (Kawashima *et al.* 2014).

### PRP18 paralogs

The two annotated PRP18 paralogs in Arabidopsis, PRP18a and PRP18b, differ in length and expression level. PRP18a is longer (420 amino acids) and ubiquitously expressed (http://www.arabidopsis.org/index.jsp; eFP Browser) while PRP18b is shorter (361 amino acids) and not expressed in the seedling material examined in this study. Most plant species examined so far have one to two copies of the PRP18 gene (https://phytozome.jgi.doe.gov/pz/portal.html#), which also differ in length in some cases, but determining whether they display different levels of expression requires more detailed analysis.

Arabidopsis PRP18a has additional sequences at the N-terminus and a recognizable PRP4 superfamily domain that are also present in PRP18a orthologs in other plant species, metazoans and *Schizosaccharomyces pombe* (fission yeast). However, the additional sequences at the N-terminus and the PRP4 domain are largely missing from Arabidopsis PRP18b and budding yeast Prp18. It has previously been suggested that PRP18b may be similar functionally to budding yeast Prp18 whereas PRP18a may have taken on added functions or capabilities endowed by the N-terminal extension (Annamalai 2011). In this context, it is interesting to note that the presence of the additional N-terminal sequences and the PRP4 domain in PRP18a and orthologous proteins is associated with organisms that carry out alternative splicing (fission yeast, plants and metazoans). One conjecture is that these regions help to promote flexibility of splicing patterns and facilitate alternative splicing in those organisms.

### General comments on forward screen

**Figure 5:**
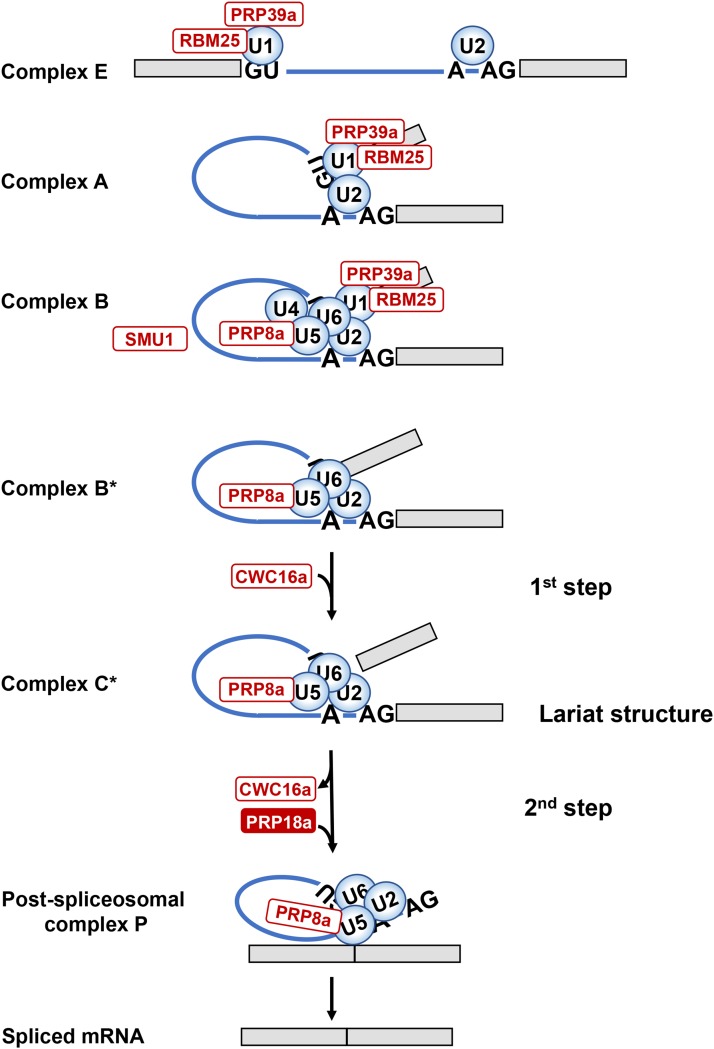
Schematic depiction of spliceosomal cycle. The main spliceosomal complexes, the two catalytic steps, and the predicted positions of factors identified in the genetic screen (white and red ovals) are shown. In *Complex E*, the U1/U2 snRNPs recognize 5′ and 3′ splice sites (GU-AG) and adenosine branch point (A) by base-pairing. In pre-spliceosomal *Complex A*, the U1 and U2 snRNPs interact to bring together 5′ and 3′ splice sites. *Complex B* (pre-catalytic spliceosome) is created by entry of preformed U4/U6.U5 tri-snRNP. Catalytic Complex B* is formed after dissociation of U1 and U4 snRNPs and other conformational and compositional changes. In complex B*, the U2, U5, and U6 snRNPs are positioned by scaffold protein PRP8a and additional proteins, including step I factor CWC16a, to execute the first step of splicing, which releases the 5′ exon and creates an intron-3′ exon lariat structure. In catalytic Complex C*, the U2, U5 and U6 snRNPs are positioned by PRP8a and additional proteins, including step II factor PRP18a, to carry out the second step of splicing, which excises the intron and joins the two exons. After formation of the *Post-spliceosomal complex P*, the spliced mRNA is released (Matera and Wang 2014). PRP39a and RBM25 are U1 snRNP components; PRP8a is a constituent of the U5 snRNP; SMU1 is present in the B complex but exits before formation of catalytic complex B*; CWC16a is a step I-specific factor; PRP18a, identified in the present study, is a step II-specific factor. Other factors not shown that were identified in the screen include: SmFa, a core snRNP protein and present in U1, U2, U4 and U5 snRNPs; coilin, which participates in snRNP maturation; and RTF2, which acts at an unknown stage of splicing.

Our forward screen, which is still ongoing, has already identified factors specific for each splicing step and for different spliceosomal complexes (Figure 5). PRP18a is the first putative step II-specific factor that we have recovered. It is still not clear why the screen has retrieved these particular splicing factors, several of which (RTF2, CWC16a, SMFa and PRP18a) had not been investigated prior to our studies. The identification of mutants defective at multiple stages of the splicing process, and often only a single member of a paralogous gene pair, hints that the screen may be tapping into a specialized splicing pathway involving a dedicated set of components. We have previously noted links to stress tolerance for coilin and SMFa, both of which were identified in this screen (Kanno *et al.* 2016, 2017a). These findings may be relevant for the known contribution of alternative splicing to plant responses to stress and environmental signals (Staiger and Brown, 2013: Filichkin *et al.* 2015). Recent cryo-EM data from budding yeast have demonstrated that the step I factor Yju2 (the ortholog of Arabidopsis CWC16 proteins) and Prp18 interact closely with Prp8 in catalytic core of the B* and C* complexes to facilitate step I and step II reactions, respectively (Galej *et al.* 2016; Wan *et al.* 2016; Yan *et al.* 2017). Our screen has identified mutants defective in all three of these splicing factors, confirming that the alternatively spliced *GFP* reporter gene system is capable of revealing central, conserved splicing proteins predicted to act directly at the catalytic site. Moreover, our findings validate a key role for these factors in pre-mRNA splicing in plants. Further analysis of these mutants and the additional, uncharacterized mutants emerging from the screen should assist in determining the mechanistic roles and interconnections of a coherent set of splicing factors, and broaden knowledge of alternative splicing in plant growth and development.

## Supplementary Material

Supplemental Material is available online at www.g3journal.org/lookup/suppl/doi:10.1534/g3.118.200022/-/DC1.
