# Supplementary material for: A Genetic Screen Identifies PRP18a, a Putative Second Step Splicing Factor Important for Alternative Splicing and a Normal Phenotype in Arabidopsis thaliana
Source: G3 (Bethesda). 2018 Feb 27;8(4):1367–77. doi: 10.1534/g3.118.200022 (PMC5873924; doi:10.1534/g3.118.200022)
Supplement: Supplementary file 1 [file 1367FigureS1.rtf]

A.thaliana_PRP18a	1 MDLLREEILKKRKSLAEESGGKKFFKRSEIEQKKIQKLREEERREHELKAQRRAAAAASG
A.thaliana_PRP18b	1 ------------------------------------------------------------
G.max			1 MDLLKQELLKKRQSLAQDTGGKKFFKRSEIQQKEIQKLREQEKRELEAKSQKRLATSSDN
P.trichocarpa      1 MDLLKQELLKKRQTLAQDTGGKKFFKRSEIQQKEIQKLREQEKRELEAKSKRQSSSSSIT
S.lycopersicum	1 MDILKQELEQRRKNLAQDVGGRKVFKRSEIEQKRLQRIHEEEKREAEAKALRQKQFEQNQ
A.tricopoda		1 MDLLKKELERKRQSVNSDFGGRKFAKRSEIEQRKLQQLREEEQNELQSKAAIKEKSAQNP
Z.mays		1 MDLLKRELEKKRKAATADFGGKSFVRRSELEQKQLQKRRDE-HRQLLAKAGTSAPSANSA
O.sativa		1 MDLLKRELEKKRKAATADFGGKSFVRRSELEQKQLQKRRHEEHRQLLSKA----PPATSS
S.bicolor		1 MDLLKRELEKKRKAATADFGGKSFVRRSELEQKQLQKRRDE-HRQLLAKASASAPSPDSA
S.moellendorffi	1 MDALKQEIERKRKQMQEEFGSRKFVKRAELEKKELEKRRREEEAAAAR---ARAAGSSKG
P.patens		1 MDALKAELERKRKATSEVSKGRKFVRRGEIDQRELERKKEQEKKELLEKAKKKAAPGTQD


A.thaliana_PRP18a	61 GDGKSSGSAPGSSNAATSASS------------------KSSASDAAAIADSKAL----T
A.thaliana_PRP18b	 1 ---MVVSKSTNSRDDNKHLGADENPNEFINSKCFK-------LASHFKTR-DFRFGQLSR
G.max			61 A-----ATAPSSSSTASAS-----------------------ASSTIASSSAASLTNEQN
P.trichocarpa	61 SS--STTTTPNSSVAASTTTT------------------TTTKNSSSTATTSKPLTVEQN
S.lycopersicum	61 KS-----TDDSSSKSNSTTDT------------------QCLKQDASSLSVSKALTDEQK
A.tricopoda		61 QVS---GQKPD-------------------------------APPSLIPSSKSQNSDEQK
Z.mays		60 AA--ATG------------------------------------------------SEERR
O.sativa		57 AS--AAGSDPSNPNADPAQSSAANPNPNSSSS-AAAASVPPGLAGKKTAQDEALLSEERR
S.bicolor		60 AA--ATGSDTSNPDAA-AQAAAANPNPSSSSSASSAPSVPPALAPKKTTQEEALLSEERR
S.moellendorffi	58 GE--VRSDDQSSER---------------------------PSASATSSKSRVNLSEDEK
P.patens		61 SS--AAGTDASVSA---------------------------PAAAKLSSKSKEVGSEEET


A.thaliana_PRP18a	 99 DENLILPRQEVIRRL-----RFLKQPMTLFGEDDQSRLDRLKYVLKEGLF-EVD-SDMTE
A.thaliana_PRP18b	 50 KDGNNFKSQRVTHRRDTQPNRGRGSNLTYSWMSFIVRLDRLKYVLKEGLFEEVD-NDMTQ
G.max			 93 IDNLVLPKPEVIRRL-----RFLKQPVTLFGEDDDARLDRLKYVLKAGVF-EVD-SDMTE
P.trichocarpa	101 IDNLVLPKQEVIRRF-----RFLKQPITLFGEDDDARLDRLKYVLKAGLF-EVD-SDMTE
S.lycopersicum	 98 IDGLNLPRQEVVRRL-----RFLKQPVTLFGEDDEARLDRLKFVLKAGTF-EVDDSDMTE
A.tricopoda		 87 IDELDLPRAEVIRRL-----RFLKQPITLFGEDDKARLDRLKLLLKAGIL-EID-NDMTE
Z.mays		 70 IDELDLPRHEVVRRL-----RVLREPVTLFGEDDDARLARFKLVLKSGVIDD---IDMTE
O.sativa		114 IDELDLPRHEVVRRL-----RILREPVTLFGEDDADRLARLKFVLKSGVIDDIDDLDMTE
S.bicolor		117 IDELDLPRHEVVRRL-----RVLREPVTLFGEDDDARLARFKLVLKSGVIDDIDDIDMTE
S.moellendorffi	 89 LDDLVLPNAEVIRRL-----RLLKQPITLFGEDDEMRLERLKVALRAGVTD-T-DSELME
P.patens		 92 LDELELPRAEVIRRL-----RYLKQPVTLFGEDDDDRLARLKALLKAGMTD-P-DSELME


A.thaliana_PRP18a	152 GQTNDFLRDIAELKKRQKSG-MMGDRKRKSRDERGRDEGDRGETR-----EDELSGGESS
A.thaliana_PRP18b	109 GETNDFLRDITELKKRERSSGLMNDRKRKTSNDDE-----------------------LI
G.max			146 GQTNDFLRDIAELRKRQKTG-ILGERKRQKADDGAAEDREGGAGD-----DDLSDCGGSD
P.trichocarpa	154 GQTNDFLRDIAELRKRQKSG-IVSGRKRKDREDGGGEDGEGGEGD-----GELGGECGAG
S.lycopersicum	152 GQTNDFLRDIVELKKRQKSG-MMSERKRKVTED-SGEDKDGGGGD-----EDLSGDGNSS
A.tricopoda		140 GQTNDFLRDMAELRQRQKSG-LLHSRKKRKEKDGVEDREGGGQGD-----DENSGDGGSS
Z.mays		122 GQTNDFLRDMIEMRKRQKAG-RDTYAKGKSKRVDGGD---WGAAGGSADDGDA--KGSGD
O.sativa		169 GQTNDFLRDMVELRKRQRAG-RDTYVKGKGKRAGGGDGGEGGAGGDNADDGDGDGRRSGD
S.bicolor		172 GQTNDFLRDMIEMRKRQKAG-RDTYAKGKGKRVGGGDGGDGGAAGDSADDGDA--KGSGD
S.moellendorffi	142 GQRNDFLMDLAELKKREKHG--LEPRKEKNKEKEDDDRGDDNAGGEALD----GES----
P.patens		145 GQRNDFLVDMAELRKREKHG-RLEPRKEKNKEKDGGDDGDRDGGGGGEQ-NITGDGGFSS


A.thaliana_PRP18a	206 DVDADKDMKRLKANFEDLCDEDKILVFYKKLLIEWKQELDAMENTERRTAKGKQMVATFK
A.thaliana_PRP18b	146 GAEKEDLKLLEEANFEDLCDEDKILVFCKKLLLEWKQELEAMENTERRTAIGKQMLATFN
G.max			200 GADADKDLKRMKANFEELCDEDKILVFFKKLLNEWKQELREMPEAEKRTAKGKSMVATFK
P.trichocarpa	208 GGDNDLDSKRMKANFEELCEEDKILVFFKRLLNEWNQELDEMAEAEKRTAKGKSMVATFK
S.lycopersicum	205 GVDHDKDLKRMKTNFVELCDEDKILVFFKKLLNEWNQELDEMTDSEKRTAKGKSMVATFK
A.tricopoda		194 GMEGDKDLKRMKSDFSELCDEDKILVFFKRLLNEWNQELEEMPEAEKRTGKGKAMVATFK
Z.mays		176 DVDADKDSKRMRTKFEELCNEDKILVFFKKLLNEWNQELDEMTELEKRTAKGKSMVATFK
O.sativa		228 DADADKDSKRMKTKFEELCDEDKILVFFKKLLIEWNQELDEMPELEKRTAKGKSMVATFK
S.bicolor		229 DADADKDSKRMRTKFEELCNEDKILVFFKKLLNEWNQELDEMTELEKRTAKGKSMVATFK
S.moellendorffi	192 GGDKDK-DLLMKANFEELCDEDKILVFFKRLIQEWEQEIKARPDAEKRGG---RIVATFK
P.patens		203 GIDNDKDVKRLKANFDELSDEDKILVFFKRLLLEWEQELENRPEAEQRTGRGKSSVATFK


A.thaliana_PRP18a	266 QCARYLVPLFNLCRKKGLPADIRQALMVMVNHCIKRDYLAAMDHYIKLAIGNAPWPIGVT
A.thaliana_PRP18b	206 QCARYLTPLFHLCRNKCLPADIRQGLMVMVNCWIKRDYLDATAQFIKLAIGNAPWPIGVT
G.max			260 QCARYLNPLFKFCRKKVLPDDIRQALLLMVECCMRRDYLAAMDHYIKLAIGNAPWPIGVT
P.trichocarpa	268 QCARYLNPLFDFSRKKILPSDIRQGLLLMVECCMRRDYLAAMDHYIRLAIGNAPWPIGVT
S.lycopersicum	265 QCARYLHPLFKFCRKKLLPDDIRQALLVVVECCMKRDYLAAMDQYIKMAIGNAPWPIGVT
A.tricopoda		254 QCARYLNPLFKFCRKKVLPDDIRQALLVVVECCMKRNYLAAMDQYIKLAIGNAPWPIGVT
Z.mays		236 QCARYLSPLFEFCRKKVLPDDIRRALLVIVECCMKRDYLAAMDQYIKLAIGNAPWPIGVT
O.sativa		288 QCARYLSPLFEFCRKQVLPDDIRQALLVIVECCMKRDYLAAMDQYIKLAIGNAPWPIGVT
S.bicolor		289 QCARYLSPLFEFCRKKVLPDDIRQALLVIVECCMKRDYLAAMDQYIKLAIGNAPWPIGVT
S.moellendorffi	248 QCSRYLKPLFKLCSKKLLPDDIRQALVIVVDCCRRRDYLAAMDQYIKLAIGNAPWPIGVT
P.patens		263 QCARYLKPLFKMCRKKILPDDIRTALMIIVKNCMERDYLTAMDQYIKLAIGNAPWPIGVT

                            prp18a-1(A334V)
↓ 


A.thaliana_PRP18a	326 MVGIHERSAREKIYTN-SVAHIMNDETTRKYLQSVKRLMTFCQRRYPTMPSKAVEFNSLA
A.thaliana_PRP18b	266 MVGIHERSAREKISTSSSVAHIMNNETTRKYLQSVKRLMTFCQRRYSALPSKSIEFNSLA
G.max			320 MVGIHERSAREKIYTN-SVAHIMNDETTRKYLQSVKRLMTFCQRRYPTLPSKAVEFNSLA
P.trichocarpa	328 MVGIHERSAREKIYTN-SVAHIMNDETTRKYLQSVKRLMTFCQRRYPTMPSKAVEFNSLA
S.lycopersicum	325 MVGIHERSAREKIYTN-SVAHIMNDETTRKYLQSVKRLMTFCQRRYPAMPSKAVEFNSLA
A.tricopoda		314 MVGIHERSAREKIYTN-SVAHIMNDETTRKYLQSVKRLMTLCQRRYPSMPSKAVEFNSLA
Z.mays		296 MVGIHERSAREKIYTN-SVAHIMNDETTRKYLQSIKRLMTLSQRRYPALPSKSVEFNSLA
O.sativa		348 MVGIHERSAREKIYTN-SVAHIMNDETTRKYLQSIKRLMTLCQRRYPALPSKSVEFNSLA
S.bicolor		349 MVGIHERSAREKIYTN-SVAHIMNDETTRKYLQSIKRLMTLCQRRYPALPSKSVEFNSLA
S.moellendorffi	308 MVGIHERSAREKIYAN-SVAHIMNDETTRKYLQSIKRLMTLCQRRYPSLPSRAVEFNSLA
P.patens		323 MVGIHERSAREKIYTN-SVAHIMNDETTRKYLQSIKRLMTLCQRRYPSMPSKSVEFNSLA


A.thaliana_PRP18a	385 NGSDLQSLLAEERFFGGNREQVSEERLRLMPSQSES*---
A.thaliana_PRP18b	326 NGSNLHSLLAEERFFAADRERVSEERLWLMPSLNEI*---
G.max			379 NGSDLHSLLAEERFSGGNQA-ASEERLRIMPAPRDS*---
P.trichocarpa	387 NGSDLQSLLAEERVFDGNQP--SEGRLRLMPAPDEN*---
S.lycopersicum	384 NGSDLQSLLAEEGTSGGSQT--SEERLRIMPA*-------
A.tricopoda		373 NGSDLQSLLSEEITNP-----SGEDRLMLMPAPKENSTHI*
Z.mays		355 NGSDLQALLSEENGSA---K-ASEERLRFMPAYAFVD*--
O.sativa		407 NGSDLQALLSEEKDPG---NPPSEDRLRLMPASKE*----
S.bicolor		408 NGSDLQALLSEENGSG---K-ASEERLRLMPASKE*----
S.moellendorffi	367 NGSDLQTLLDEEQKLLEGTH--HQS*--------------
P.patens		382 NGSDLKSLLTEEKNKTGSVA--AEERLRLLAARDEDR*--

Figure S1: Amino acid sequence alignments of PRP18 proteins in selected plant species
Alignments were carried out using Clustal Omega (http://www.ebi.ac.uk/Tools/msa/clustalo/) and the resulting alignments were processed by BoxShade (http://www.ch.embnet.org/software/BOX_form.html).
The position of the prp18a-1 mutation, A334V, is indicated (red letters). This alanine residue is conserved in all plant species shown. Note the missing 60 amino acids in the N-terminus and the non-homologous PRP4 motif region in Arabidopsis PRP18b compared to PRP18a proteins in Arabidopsis and other plants. NCBI ID numbers for the sequences shown; G. max; XP_003521247; P. trichocarpa; XP_002307588; S. lycopersicum; XP_004247169; A. tricopoda; XP_006844663; Z. mays; XP_008663390; O. sativa; XP_015647118; S. bicolor; XP_002463061; S. moellendorffi; XP_002964125; P. patens; XP_001782842
