# Supplementary material for: A Genetic Screen Identifies PRP18a, a Putative Second Step Splicing Factor Important for Alternative Splicing and a Normal Phenotype in Arabidopsis thaliana
Source: G3 (Bethesda). 2018 Feb 27;8(4):1367–77. doi: 10.1534/g3.118.200022 (PMC5873924; doi:10.1534/g3.118.200022)
Supplement: Supplementary file 2 [file 1367FigureS2.rtf]

A.thaliana		1 -----MDLLREEILKKRKSLAEE---SGGKKFFKRSEIEQKKIQKLREEERREHELKAQR
H.sapiens		1 -----MDILKSEILRKRQLVEDRNLLVENKKYFKRSELAKKEEEAYFERCGY----KIQP
M.musculus		1 -----MDILKSEILRKRQLVEDRNLLVENKKYFKRSELARKEEEAYYERCGY----KIQP
D.melanogaster	1 -----MDILKAEIARKRKLLEQRQLVDEKKKYFRRGDLNAKNTEEVLQKVGY----IKQE
C.elegans		1 --MDFKAKLLAEMAKKRKAVSGMEVKEGNAKFVKGADLEMKRNQEYERKQQEI---ASKK
S.pombe		1 -----MDFLKEEIERKRRQLEGTSELP-VKKAFRRGDWEKEREKKYLQEKQQKDEQRELK
S.cerevisiae 	1 MDLDLASILKGEISKKKKELANSK------------------------------------


A.thaliana		53 -R------------AAAAA--SGGDGKSSGSAPGSSNAATSAS--SKSSASDAAAIADSK
H.sapiens		52 -K------------E---------------E-----------D--QKPLTSSNPVLELEL
M.musculus		52 -K------------E---------------D-----------D--QKPLTSSNPVLELEL
D.melanogaster	52 -S------------V---------------E-----------A--QGQTTEGAYSF----
C.elegans		56 -R------------K--------VDD----EIL--------QE--SSSRTKPAPVE----
S.pombe		55 KRKLEEERLKYEEKKLRISRLANKESSRNEELLTETTTPSPAVKASPASTKLSVSENDRL
S.cerevisiae	25 ------------------------------------------------------------


A.thaliana		 96 AL-TDENLILPRQEVIRRLRFLKQPMTLFGEDDQSRLDRLKYVLKEGLFEVDSDMTEGQT
H.sapiens		 71 AE-EKLPMTLSRQEVIRRLRERGEPIRLFGETDYDAFQRLRKIEI-----LTPEVNKGLR
M.musculus		 71 AE-EKLPMTLSRQEVIRRLRERGEPIRLFGETDYDAFQRLRKIEI-----LTPEVNKGLR
D.melanogaster	 67 -V-ADGQNILPRTEVIRRLRERGEPILIFGETEPEAFDRLRQCEI-----SQPEANRGFR
C.elegans		 77 NE-SEIDEKTPMSEIQTRLRQRNHPIMLFGETDIDVRKRLHQLEL-----AQPDLNEGWE
S.pombe		115 SIPEITKDNLTLTEIIAKLREMKEPIRLFGESEEATIQRYYSLLKYKKL-------EEIE
S.cerevisiae	 25 -------------------------------------------------GVQPPCTEKFQ


A.thaliana		155 NDFLRDIAELKKRQKSGMMGDRKRKSRDERGRDEGDRGETR--EDELS-GGESS--DVDA
H.sapiens		125 NDLKAALDKIDQQYLNEIVGGQEPGEEDTQN-------------DLKV-HEENT--TIEE
M.musculus		125 NDLKAALDKIDQQYLNEIVGGQEPGEEDTQN-------------DLKV-HEENT--TIEE
D.melanogaster	120 NDFQEAMEQVDAAYLQEMFANTPTTKEDKKS-------------DFAE-LDESV--SWES
C.elegans		131 NELQTAMKVIGKEMDKAVVEGTADSATRHD-------------IALPQ-GYEED--NWKN
S.pombe		168 NELLT------------------------------------------K-GVETI--DFEH
S.cerevisiae	 36 PHESANIDETPRQVEQE--------STDEENLSDNQSDDIRTTISKLENRPERIQEAIAQ


A.thaliana		210 DKDM-----KRLKANFED-LCDEDKILVFYKKLLIEWKQELDAMENTERRTAKGKQMVAT
H.sapiens		169 LEAL-----GESLGKGDD-HKDMDIITKFLKFLLGVWAKELNAREDYVKRSVQGKLNSAT
M.musculus		169 LEAL-----GESLGKGDD-HKDMDIITKFLKFLLGVWAKELNAREDYVKRSVQGKLNSAT
D.melanogaster	164 IQTM-----AANMGRNK--DYDMDVIITLLTFLLKLWNDQIANYSKHEKMSTKVKMTRVI
C.elegans		175 IEHN-----STLLSVDDDLKRDCDIILSICRYILARWAKDLNDRPLDVKKTAQGMHEAAH
S.pombe		183 ATTT-----K---------PKVSKQVVAFLQHGIRIWDNFLSSKSINSFESSESQMQLKI
S.cerevisiae	 88 DKTISVIIDPSQIGSTEGKPLLSMKCNLYIHEILSRWKASLEAYHP------------EL


A.thaliana		264 FKQCARYLVPLFNLCRKKGLPADIRQALMVMVNH-CIKRDYLAAMDHYIKLAIGNAPWPI
H.sapiens		223 QKQTESYLRPLFRKLRKRNLPADIKESITDIIKF-MLQREYVKANDAYLQMAIGNAPWPI
M.musculus		223 QKQTESYLRPLFRKLRKRNLPADIKESITDIIKF-MLQREYVKANDAYLQMAIGNAPWPI
D.melanogaster	217 YTQTKEYVKPLFRKLKHHTLPEDILDSLRDICKH-LLNRNYITASDAYLEMAIGNAPWPI
C.elegans		230 HKQTMMHLKSLMTSMERYNCNNDIRHHLAKICRLLVIDRNYLEANNAYMEMAIGNAPWPV
S.pombe		229 FRQAKQDLDVLIQLIVDEALNDDIFKSIAEICYR-CQKHEFVKANDMYLRLTIGNAPWPI
S.cerevisiae	136 FLDTKKALFPLLLQLRRNQLAPDLLISLATVLYHLQQPKEINLAVQSYMKLSIGNVAWPI


prp18a-1(A334V)
↓ 


A.thaliana		323 GVTMVGIHERSAREKIYT-NSVAHIMNDETTRKYLQSVKRLMTFCQRRYPTMPSKAVEFN
H.sapiens		282 GVTMVGIHARTGREKIFS-KHVAHVLNDETQRKYIQGLKRLMTICQKHFPTDPSKCVEYN
M.musculus		282 GVTMVGIHARTGREKIFS-KHVAHVLNDETQRKYIQGLKRLMTICQKHFPTDPSKCVEYN
D.melanogaster	276 GVTMVGIHARTGREKIFS-KNVAHVMNDETQRKYIQGLKRLMTKCQEYFPTDPSKCVEYV
C.elegans		290 GVTRSGIHQRPGSAKSYV-SNIAHVLNDETQRKYIQAFKRLMTKMQEYFPTDPSKSVEFV
S.pombe		288 GVTMVGIHERSAHQRLQA-NPSSNILKDEKKRKCLQALKRFITFQERESSNLPEYTD---
S.cerevisiae	196 GVTSVGIHARSAHSKIQGGRNAANIMIDERTRLWITSIKRLITFEEWYTSNHDSLA----


A.thaliana		382 SLANGSDLQSLLAEERFFGGNREQVSEERLRLMPSQSES*
H.sapienw		341 AL--------------------------------------
M.musculus		341 AL--------------------------------------
D.melanogaster	335 SKKDRE----------------------------------
C.elegans		349 KKAV------------------------------------
S.pombe		     ----------------------------------------
S.cerevisiae	     ----------------------------------------


Figure S2: Amino acid alignments of PRP18 proteins in model organisms
Alignments were carried out using Clustal Omega (http://www.ebi.ac.uk/Tools/msa/clustalo/) and the resulting alignments were processed by BoxShade (http://www.ch.embnet.org/software/BOX_form.html).
The prp18a-1 mutation identified in this screen is A334V (red letters). Although this alanine residue is conserved in all plants examined (Figure S1) and in budding and fission yeasts (S. cerevisiae and S. pombe, respectively), it is substituted by a glycine in the metazoan species shown here. Alanine is a small amino acid, second only to glycine on size, and hence this constitutes a conservative change. Note the missing sequences close to the N-terminus and lack or PRP4 domain in Prp18 of S. cerevisiae. NCBI ID numbers for the sequences shown; H.sapiens; NP_003666; M.musculus; NP_080321; D.melanogaster; NP_650776; C.elegans; NP_501776; S.cerevisiae; NP_011520; S.pombe; NP_588457
