# Supplementary material for: A Genetic Screen Identifies PRP18a, a Putative Second Step Splicing Factor Important for Alternative Splicing and a Normal Phenotype in Arabidopsis thaliana
Source: G3 (Bethesda). 2018 Feb 27;8(4):1367–77. doi: 10.1534/g3.118.200022 (PMC5873924; doi:10.1534/g3.118.200022)
Supplement: Supplementary file 3 [file 1367FigureS3.pptx]

## Slide 1
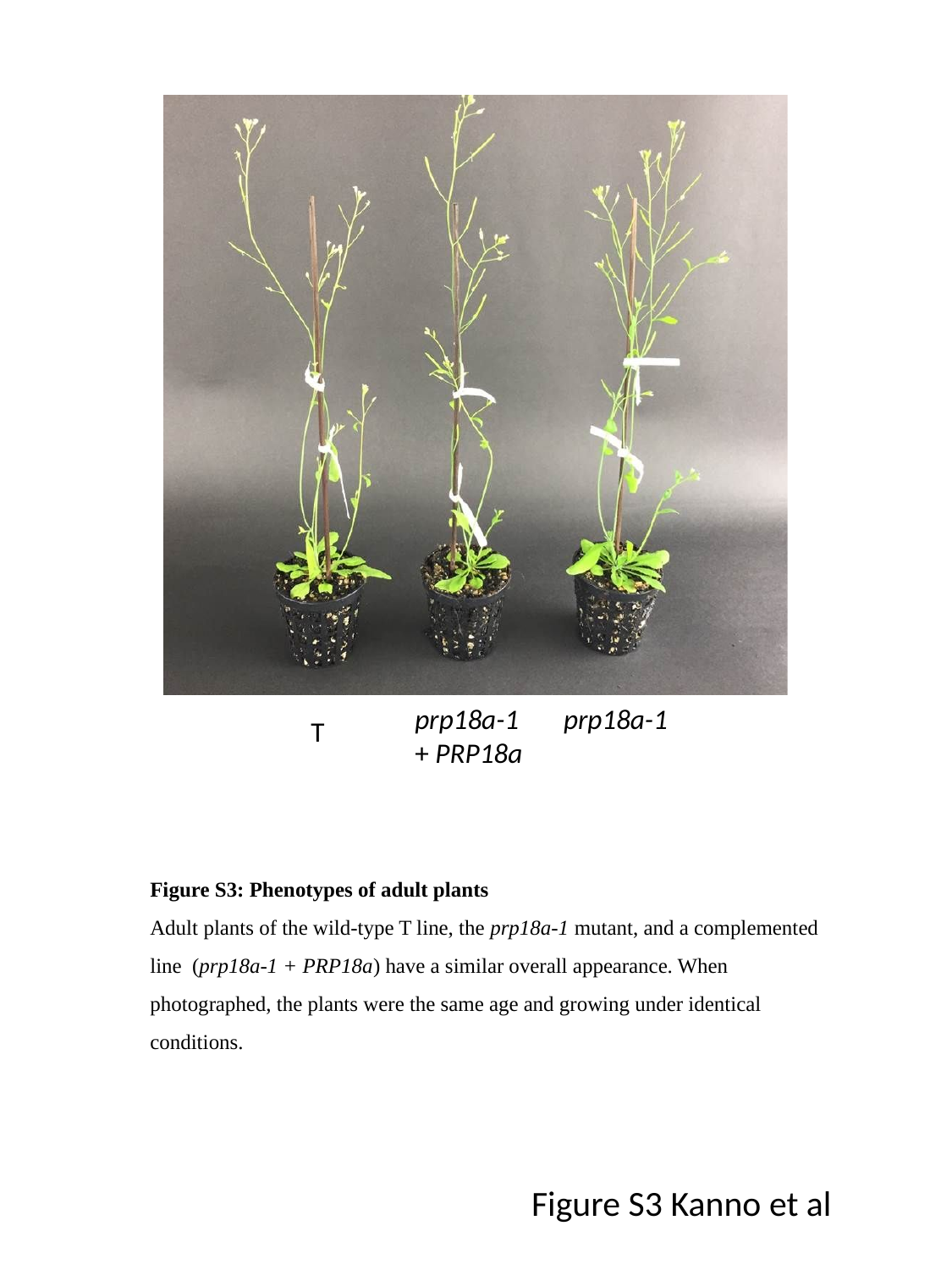

prp18a-1
prp18a-1
+ PRP18a
T
Figure S3: Phenotypes of adult plants
Adult plants of the wild-type T line, the prp18a-1 mutant, and a complemented line (prp18a-1 + PRP18a) have a similar overall appearance. When photographed, the plants were the same age and growing under identical conditions.
Figure S3 Kanno et al
