# Supplementary material for: A Genetic Screen Identifies PRP18a, a Putative Second Step Splicing Factor Important for Alternative Splicing and a Normal Phenotype in Arabidopsis thaliana
Source: G3 (Bethesda). 2018 Feb 27;8(4):1367–77. doi: 10.1534/g3.118.200022 (PMC5873924; doi:10.1534/g3.118.200022)
Supplement: Supplementary file 4 [file 1367FigureS4.pptx]

## Slide 1
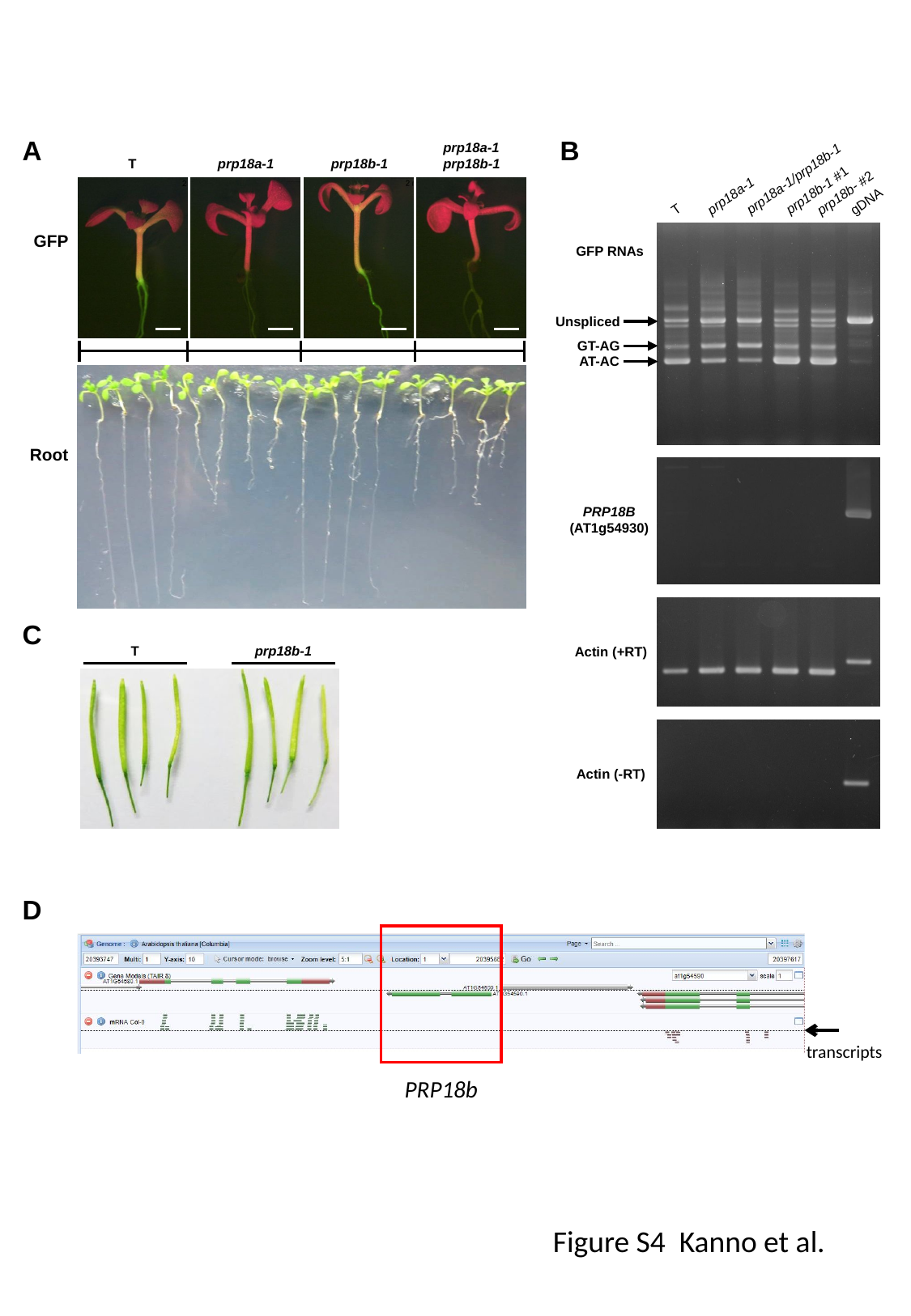

A
B
prp18a-1
prp18b-1
T
prp18a-1
prp18b-1
prp18a-1/prp18b-1
prp18b-1 #1
prp18b- #2
prp18a-1
gDNA
T
GFP RNAs
Unspliced
GT-AG
AT-AC
PRP18B
(AT1g54930)
Actin (+RT)
Actin (-RT)
GFP
Root
C
T
prp18b-1
D
transcripts
PRP18b
Figure S4 Kanno et al.

## Slide 2
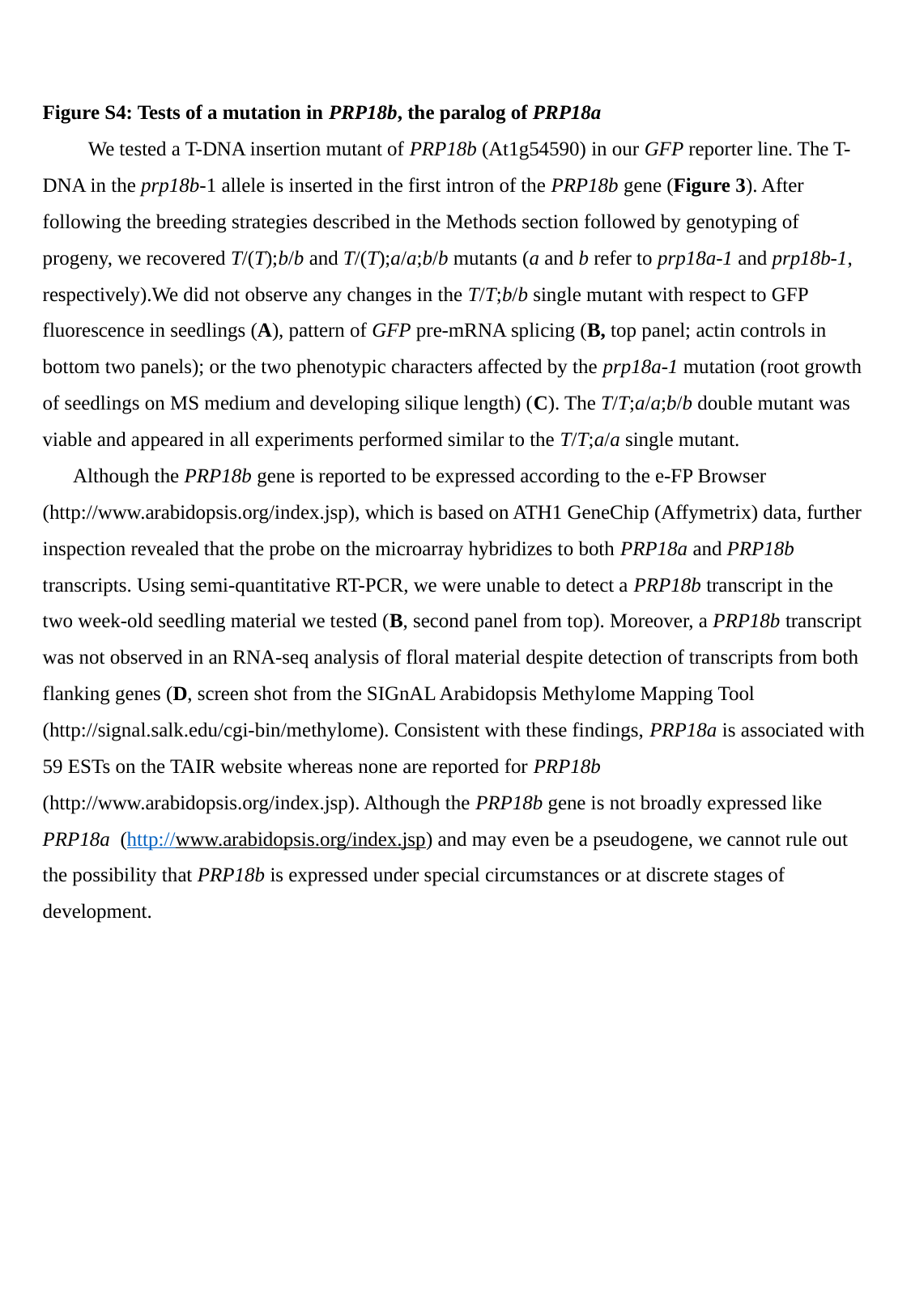

Figure S4: Tests of a mutation in PRP18b, the paralog of PRP18a
 We tested a T-DNA insertion mutant of PRP18b (At1g54590) in our GFP reporter line. The T-DNA in the prp18b-1 allele is inserted in the first intron of the PRP18b gene (Figure 3). After following the breeding strategies described in the Methods section followed by genotyping of progeny, we recovered T/(T);b/b and T/(T);a/a;b/b mutants (a and b refer to prp18a-1 and prp18b-1, respectively).We did not observe any changes in the T/T;b/b single mutant with respect to GFP fluorescence in seedlings (A), pattern of GFP pre-mRNA splicing (B, top panel; actin controls in bottom two panels); or the two phenotypic characters affected by the prp18a-1 mutation (root growth of seedlings on MS medium and developing silique length) (C). The T/T;a/a;b/b double mutant was viable and appeared in all experiments performed similar to the T/T;a/a single mutant.
 Although the PRP18b gene is reported to be expressed according to the e-FP Browser (http://www.arabidopsis.org/index.jsp), which is based on ATH1 GeneChip (Affymetrix) data, further inspection revealed that the probe on the microarray hybridizes to both PRP18a and PRP18b transcripts. Using semi-quantitative RT-PCR, we were unable to detect a PRP18b transcript in the two week-old seedling material we tested (B, second panel from top). Moreover, a PRP18b transcript was not observed in an RNA-seq analysis of floral material despite detection of transcripts from both flanking genes (D, screen shot from the SIGnAL Arabidopsis Methylome Mapping Tool (http://signal.salk.edu/cgi-bin/methylome). Consistent with these findings, PRP18a is associated with 59 ESTs on the TAIR website whereas none are reported for PRP18b (http://www.arabidopsis.org/index.jsp). Although the PRP18b gene is not broadly expressed like PRP18a (http://www.arabidopsis.org/index.jsp) and may even be a pseudogene, we cannot rule out the possibility that PRP18b is expressed under special circumstances or at discrete stages of development.
