# Supplementary material for: A Genetic Screen Identifies PRP18a, a Putative Second Step Splicing Factor Important for Alternative Splicing and a Normal Phenotype in Arabidopsis thaliana
Source: G3 (Bethesda). 2018 Feb 27;8(4):1367–77. doi: 10.1534/g3.118.200022 (PMC5873924; doi:10.1534/g3.118.200022)
Supplement: Supplementary file 9 [file 1367TableS5.docx]

**Supplementary Table 5. Primers (5’ to 3’)** Primers used in this study are listed below.

**Sequencing primers of PRP18a gene (At1g03140)**

prp_18_1 AGCTGAATCTACGTATCGTC Fragment amplification

prp_18_2 CATGCCACATAAGACTAGAC Fragment amplification

prp_18_3 CGCAAGAAATCATTCGTCTG sequencing

prp_18_4 TTCGGAGAAGATGATCAATC sequencing

prp_18_5 GGTTCCATTAGTTTGTGTAC sequencing

**PCR Primers for Genotyping**

*prp18a-1* (CAPS: *Xho*I)

prp_18_2 CATGCCACATAAGACTAGAC

prp_18_5 GGTTCCATTAGTTTGTGTAC

SALK_024667C (*prp18b-1*: T-DNA insertion)

PRP18B_F AAACTGGTCAGAACAGTGTG

PRP18B_R CGGAATTTCGAAGGTCGGAG

LBa1 TGGTTCACGTAGTGGGCCATCG

**RT-PCR**

Spreading5’ AGGCTGCATCTTCAGGCATC GFP splice variants

egfp3’ TTTACTTGTACAGCTCGTCC GFP splice variants

actin-f GCCATCCAAGCTGTTCTCTC Actin control

actin-r GGGCATCTGAATCTCTCAGC Actin control
